# Evaluating the Interrater Agreement and Acceptability of a New Reference Tool for Assessing Respiratory Rate in Children under Five with Cough and/or Difficulty Breathing

**DOI:** 10.1093/tropej/fmab046

**Published:** 2021-06-13

**Authors:** Ann-Sophie Stratil, Charlotte Ward, Tedila Habte, Alice Maurel, Max Antson, Elina Naydenova, Kevin Baker

**Affiliations:** 1 Malaria Consortium, E2 9DA, London, UK; 2 Feebris Ltd, E2 8AA, London, UK

**Keywords:** respiratory rate, pneumonia, diagnostic techniques, respiratory system, reproducibility of results, reference standards, validation study

## Abstract

**Background:**

Manual assessment of respiratory rate (RR) in children is unreliable, but remains the main method to diagnose pneumonia in low-resource settings. While automated RR counters offer a potential solution, there is currently no gold standard to validate these diagnostic aids. A video-based reference tool is proposed that allows users to annotate breaths and distortions including movement periods, allowing the exclusion of distortions from the computation of RR measures similar to how new diagnostic aids account for distortions automatically. This study evaluated the interrater agreement and acceptability of the new reference tool.

**Methods:**

Annotations were based on previously recorded reference videos of children under five years old with cough and/or difficulty breathing (*n* = 50). Five randomly selected medical experts from a panel of ten annotated each video. RR measures (breaths per minute, bpm) were computed as the number of annotated certain breaths divided by the length of calm periods after removing annotated distorted periods.

**Results:**

Reviewers showed good interrater agreement on continuous RR {standard error of measurement (SEM) [4.8 (95%CI 4.4–5.3)]} and substantial agreement on classification of fast breathing (Fleiss kappa, *κ* 0.71). Agreement was lowest in the youngest age group [< 2 months: SEM 6.2 (5.4–7.4) bpm, *κ* 0.48; 2–11 months: 4.7 (4.0–5.8) bpm, *κ* 0.84; 12–59 months: 2.6 (2.2–3.1) bpm, *κ* 0.8]. Reviewers found the functionalities of the tool helpful in annotating breaths, but remained uncertain about the validity of their annotations.

**Conclusions:**

Before the new tool can be considered a reference standard for RR assessments, interrater agreement in children younger than 2 months must be improved.

## INTRODUCTION

Pneumonia is the leading infectious cause of death in children accounting for 15% of all deaths of children under five years old in 2017 [1]. These deaths can be prevented by providing early diagnosis to suspected cases and appropriate treatment to confirmed cases depending on cause and case severity. According to the World Health Organizations (WHO) guideline for the Integrated Management of Childhood Illnesses (IMCI), primary diagnostic criteria for pneumonia are the presence of cough or difficulty breathing and a raised respiratory rate (RR) in dependence of a child’s age. In low-resource settings, RR is currently assessed by community health workers (CHWs) manually counting breaths of suspected cases [2]. While the IMCI guideline recommends CHWs counting breaths for one full minute in calm children, under real-life circumstances, younger children seldom remain calm for a full minute making it challenging for CHWs to accurately identify and count breaths [3]. Inaccurate RR assessments are common and can result in misdiagnosis of suspected pneumonia with potentially fatal consequences due to inappropriate or no treatment [4,5]. New, automated RR counters offer a potential solution by accounting for movement periods automatically or deriving RR through indirect methods not influenced by movement, thereby supporting the accurate assessment of RR under real-life circumstances [6, 7]. To introduce these new diagnostic aids at scale, their accuracy and reliability must first be validated against a robust reference standard.

A review of RR tools in 2018 found that the most commonly used reference tool in previous validation studies of new RR assessments diagnostic aids was manual counting among other methods like thoracoscopic talc insufflation and electrocardiogram [7]. However, such reference tools were often not validated against a gold standard, limiting the validity of the results pertained from these studies [7, 8]. Using manual counting as a reference standard for new diagnostic aids is agreed to be particularly challenging as new and automated tools are developed to improve the reliability and accuracy of manually counting breaths, hence making it an improper reference. There is need for a gold standard method to facilitate the validation and comparability of the performance of new diagnostic aids [9].

Recently, a new reference tool was developed which facilitates manual annotation of breaths based on videos. This new tool expands on the commonly used manual count reference by allowing reviewers to not only annotate breaths but also the certainty of a breath and distortions including non-breath movements and other interruptions to the normal breathing such as crying. Distorted periods derived from time points annotated as distortions can then be excluded from RR measures, similar to how new diagnostic aids account for distortions automatically. This functionality makes it a potential candidate for a new reference standard that assesses the accuracy and reliability of new automated diagnostic aids. However, the accuracy and reliability of the new tool itself still needs to be validated.

This study aimed to evaluate the interrater agreement on RR measures between medical professionals using this new tool and the perceived acceptability of the tool for assessing RR in children under five years old with cough and/or difficulty breathing.

## METHODS

### Study design and setting

Interrater agreement between RR measures of reviewers using the new reference tool was investigated. Annotations were based on videos that had been previously recorded for research on RR assessments of children under five years old presenting with cough and/or difficulty breathing. Five randomly selected medical experts from panel of ten annotated each reference video. Acceptability of the new tool to users was explored through focus group discussions (FGDs).

The study was conducted in Hawassa, Southern Nations, Nationalities and Peoples' Region (SNNPR), Ethiopia between April and September 2019.

### Study sample

#### Sample size

The reliability measure intraclass correlation coefficient (ICC) was used as a basis for the sample size calculation. The primary outcome standard error of measurement (SEM) used in this study can be derived directly from ICC. Following the method proposed by Bonett [10] for estimating ICC with desired precision, 51 videos to be assessed by five reviewers each were required to detect a point estimate of 0.7 (in line with the outcomes of a previous study [11]) within ± 0.1 of its true value and a type I error of 5%.

#### Selection criteria and sampling methods

Reviewers annotated videos that had been collected in two previous studies in Ethiopia, Uganda and South Sudan [12, 13]. During these studies, children had been included if they were 0–59 months old and presented with cough and/or difficulty breathing, but did not show any general danger signs [2]. Video quality was assured by including videos where full chest and belly of the child were visible, the video was sufficiently bright and free from external distractions and the camera remained still for the duration of the video. Videos with > 30 s of distortion (e.g. when child moved, hiccupped or cried) were excluded as these did not meet the IMCI criteria that during manual RR count, the counter should ‘ensure the child is calm’. Videos fulfilling these eligibility criteria were randomly selected via stratified random sampling by source study. Selection was balanced between clinically relevant age groups < 2, 2–11 and 12–59 months.

A panel of ten reviewers was recruited through newspaper advertisements using the following criteria: medical staff with at least two years’ paediatric experience, experience in manually counting breaths for RR assessments, English proficiency and basic knowledge on computer use. For each video to be reviewed, a random set of five reviewers was selected from the larger panel by simple random sampling using a web-based random number generator. At the end of the study, all reviewers were invited to participate in FGDs.

### Data collection

#### Video annotations

**Fig. 1.**
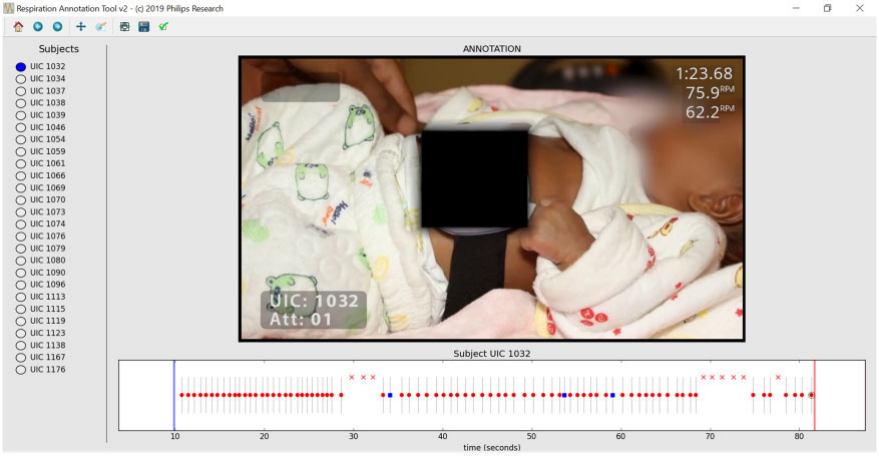
Timeline of video annotation. Red dots are annotated certain breaths, blue squares are annotated uncertain breaths and red Xs are moments of distortions.

All reviewers received four days training on using the reference tool and two assessments were conducted prior to data collection. A manual RR counting test was conducted using an established WHO RR training video [14]. All ten reviewers were within ± 3 breaths per minute (bpm) of each other after two counting attempts. They then conducted an annotation test using the software and all reviewers were within ± 3 bpm of the RR obtained by the developer of the reference tool. Reviewers differentiated annotations (time points) between certain breaths, uncertain breaths and distortions and were instructed to do so in successive video review rounds. Certain and uncertain breaths were marked at the (perceived) time point of maximum inspiration within a breath cycle. Uncertain breaths were defined as very shallow, incomplete or difficult to judge breath movements, while distortions were defined as non-breath movements including interruptions to the normal breathing such as crying. Reviewers were encouraged to use support functionalities including changing playback speed and zoom levels, adjusting brightness and moving back and forth along the video timeline to support their judgment. They independently timed the duration they spent on each video. Annotation data containing the type and time stamp of each annotation along the video timeline were extracted in tabular form for analysis. Figure 1 shows the timeline of a video annotation where red dots are annotated certain breaths, blue squares are annotated uncertain breaths and annotated Xs are moments of distortions.

#### Qualitative data

Two FGDs with five reviewers each were conducted to collect qualitative data on acceptability of the reference tool among reviewers. The topic guides for assessment were developed using a comprehensive conceptual framework of acceptability of healthcare interventions [15]. Discussions were facilitated in Amharic by a trained interviewer. Discussions were audio-recorded, transcribed verbatim and subsequently translated to English for analysis.

### Computation of RR measures

**Fig. 2.**
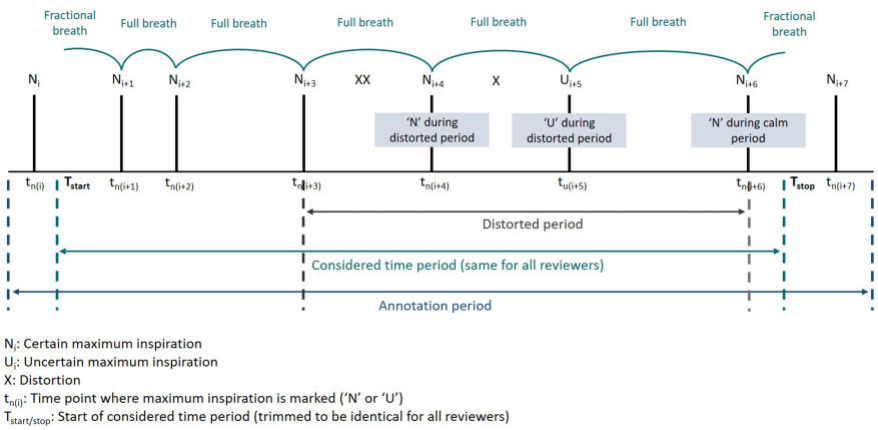
Schematic annotation pattern.

RR measures were computed using Python 3. RR (bpm) was computed as the number of certain breath cycles during calm periods after removal of distorted periods divided by the total duration of calm periods in seconds and multiplied by 60. A full breath cycle is usually defined as the time from the beginning of inhalation to the end of exhalation. As reviewers marked time points of maximum inspiration, in this study, a breath cycle was defined as the time period between two certain breath annotations. The time period between these two points of maximum inspiration included the exhalation and inhalation period hence depicts the time period for a full breath cycle. Fractional breaths at the beginning or end of the calm period were included as proportions of a breath cycle. For all reviewers, the exact same time period was considered to obtain calm periods. See Fig. 2 for a schematic annotation pattern. The Python code for RR computation is referenced under ‘Availability of data and materials’. Fast breathing classification was determined by age-based cut-offs according to IMCI guidelines [2]. Time taken was defined as the period during which a reviewer watched and annotated a video. Breaks between review cycles were not included.

### Analysis

#### Interrater agreement

Data were analysed using STATA13 (StataCorp. 2013, Stata Statistical Software: Release 13. StataCorp LP., College Station, TX, USA) and Microsoft Excel 2016 (version 16.0.5095.1000).

SEM and its 95% confidence interval (95% CI) were computed following the methodology outlined by Stratford and Goldsmith [16]. Secondary measures of agreement quantifying agreement on classification on fast breathing (normal vs. fast based on IMCI guideline criteria) were Fleiss’ kappa where *κ* < 0 constituted poor, 0.01–0.20 slight, 0.21–0.40 fair, 0.41–0.60 moderate, 0.61–0.80 substantial and 0.81–1.00 almost perfect agreement [17]; and proportion of videos where all reviewers agreed on presence of fast breathing. Associations between categorical variables were investigated with Kruskal–Wallis test with Holm’s adjustment and Dunn’ multiple comparison test. Missing data were handled through pairwise deletion.

#### Acceptability

Thematic analysis of the qualitative data was conducted using MAXQDA (VERBI Software, Berlin, Germany, 2016) and Microsoft Excel (2016). Transcripts were coded against an agreed set of codes by two research team members independently. These initial codes were then consolidated into broader themes [18].

### Ethical approval and consent

Ethical approval for the study was provided by the SNNPR Regional State Health Bureau Health Research Ethics Committee (PN6/9/32080). Informed consent was provided by all participants of this study.

## RESULTS

### Study population

**Fig. 3.**
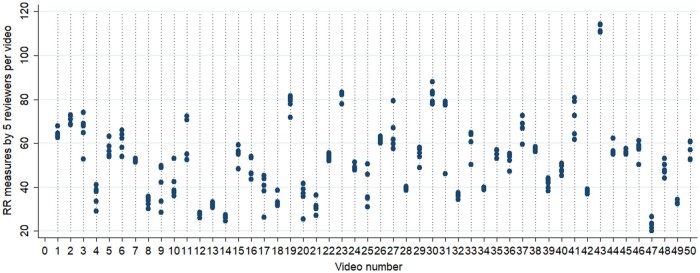
Respiratory rate measures by five reviewers across 50 videos.

Ten reviewers were recruited for the study. Notably, the majority (80%, 8/10) was male and on average 30 [standard deviation (SD) 4] years old with an average of 7.6 (SD 2.3) years of work experience in their role. All reviewers worked either in health centres or in hospitals and held a degree in Nursing or Public Health (Fig. 3).

Over half (56%, 28/50) of children in the video sample were male, just over a third (38%, 19/50) were in the youngest age group (< 2 months) and the majority of videos (78%, 39/50) had been recorded in Ethiopia (Table 1).

**Table 1:** Characteristics of video sample

Characteristics	Analysed population (*n* = 50^a^)
Gender	
Male	28 (56%)
Female	22 (44%)
Age group	
<2 months	19 (38%)
2–11 months	14 (28%)
12–59 months	17 (34%)
Country where video was recorded	
Ethiopia	39 (78%)
Uganda	9 (18%)
South Sudan	2 (4%)

a 51 videos included initially, but annotation data could not be retrieved for *n* = 1.

#### Annotation measures

The mean video length (considered time period) was 66.1 (SD 14.9) s. Mean time taken by reviewers to annotate a video was 29.7 (SD 11.1) times the duration of the considered period. Mean distorted period per video was 19.5 (SD 14.3) % of the considered period. Overall average computed RR was 52.0 (SD 17.2) bpm with decreasing average RR from younger to older age groups (Table 2).

**Table 2:** Annotation measures

	*N*	Mean (SD)
Considered time period	50	66.1 (14.9) s
Time taken to annotate video	50	29.7 (11.1) times the considered period
Distorted period per video	50	19.5 (14.3)% of the considered period
Highest proportion distorted (≥ 23.0%)	16	37.6 (7.9)%
Medium proportion distorted (> 10.0–< 23.0%)	17	16.4 (3.8)%
Shortest proportion distorted (≤ 10.0%)	17	5.4 (2.4)%
Respiratory rate	50	52.0 (17.2) bpm
< 2 months	19	61.2 (16.3) bpm
2–11 months	14	56.2 (14.6) bpm
12–59 months	17	38.2 (10.7) bpm

### Interrater reliability and agreement

The overall SEM was 4.8 (95% CI 4.4–5.3) bpm. Interrater agreement on fast breathing was substantial (*κ* 0.71). This corresponded to all five reviewers agreeing on classification of fast breathing for 70% (35/50) of the videos. Agreement measures differed substantially between age groups. In the youngest age group (< 2 months), SEM was 6.2 (5.4–7.4) bpm and agreement on fast breathing was moderate (*κ* 0.48), whereas in the older age groups (2–11 and 12–59 months), SEM was 4.7 (4.0–5.8) bpm and 2.6 (2.2–3.1) bpm and agreement on fast breathing was almost perfect (*κ* 0.84) and substantial (*κ* 0.8), respectively. Exploratory analysis indicated that agreement between five reviewers seemed to be higher in videos where reviewers annotated less distortion. Age group of the child and distortion annotated by reviewers seemed to be correlated: Videos of the children 12–59 months showed a significantly lower degree of distortion than videos of children < 2 months (*χ*^2^ 2.090, *p* 0.04) and 2–11 months (*χ*^2^ 2.227, *p* 0.04) (Table 3).

**Table 3:** Interrater reliability and agreement measures

	*N*	SEM (95% CI)	Kappa for agreement on fast breathing	Videos with agreement on fast breathing, *n* (%)
Total	50	4.8 (4.4–5.3) bpm	0.71— substantial	35 (70%)
Age groups				
< 2 months	19	6.2 (5.4–7.4) bpm	0.48 — moderate	9 (47%)
2–11 months	14	4.7 (4.0–5.8) bpm	0.84 — almost perfect	12 (86%)
12–59 months	17	2.6 (2.2–3.1) bpm	0.80 — substantial	14 (82%)
Average distorted period per video (% of considered period)				
Highest proportion distorted (≥ 23.0%)	16	7.3 (6.3–8.9) bpm	0.57 — moderate	10 (63%)
Medium proportion distorted (> 10.0–< 23.0%)	17	3.8 (3.3–4.6) bpm	0.64 — substantial	11 (65%)
Shortest proportion distorted (≤ 10.0%)	17	1.7 (1.5–2.1) bpm	0.86 — almost perfect	14 (82%)

### Acceptability

FGDs among reviewers on the acceptability of the reference tool centred around the following themes: (i) benefits of the tool; (ii) limitations of the tool; and (iii) overall acceptability.

Reviewers noted that tool functionalities, like slowing down, changing colour adjusting brightness helped them in distinguishing these different kinds of breaths: ‘By changing colour, we can see whether the movement is normal breath or shallow or distortion’.

However, videos with lots of distortions, movements and uncertain breaths were considered difficult to annotate with videos of younger children being harder to annotate in general. Reviewers found it challenging to find the exact time to mark the start/end of distortion, to not miss shallow breaths between distortions or to identify breaths in restless and crying children. One reviewer noted the challenges that indirectly observing children entails: ‘It was difficult to me to mark because you can’t calm children as [an] actual patient’, while another detailed that ‘when [the] child is restless and crying, the abdomen becomes rigid and breathing can’t be seen’. One reviewer pointed out age and case severity as factors that complicate the assessment: ‘It depends on child’s age, stability and severity of disease. If [a] child is severely sick, RR increases and marking many breathes is time consuming. I remember a video took 63 min from me’. In summary, while tool functionalities like slowing down or adjusting brightness helped identifying difficult breaths, using these functions remained challenging and time consuming.

While some reviewers welcomed the additional functionality of this tool being able to mark distorted periods, others suggested they would only trust tool under certain conditions or suggested amendments calling for a revision of the tool that excluded distortion periods from videos or automated the detection of distorted periods. These concerns indicate that, while the tool provided the helpful functionalities to annotate breaths and distorted periods, reviewers continued to feel uncertain about their annotations.

## DISCUSSION

Here, we presented results on the interrater agreement of a new reference tool for RR-measuring diagnostic aids. Overall, the tool showed good interrater agreement between reviewers on continuous RR measures [SEM 4.8 (4.4–5.3) bpm] and substantial agreement between five reviewers on the classification of fast breathing (*κ* 0.71).

These results are in line with previous studies assessing interrater agreement of direct manual count or video-based count to obtain RR. Another study in north-eastern Tanzania measuring the agreement between two paediatricians reviewing RR videos of children aged 2–59 months found similar levels of interrater agreement on classification of fast breathing (*κ* 0.85) [19]. Findings from the ARIDA study that included a subset of the videos used in this study also found similar agreement between two experts counters assessing RR through manual count using the Mark 2 ARI timer (SEM 3.9 bpm, *κ* 0.83) and based on counting from videos without annotation software (SEM 6.6 bpm, *κ* 0.86) [11].

However, in children younger than 2 months and in videos with high distortion, interrater agreement was substantially lower than the overall result. Agreement on the classification of fast breathing was only moderate for videos of children younger than < 2 months and for videos with highest average distortion. This is supported by the qualitative assessments where reviewers reported that the videos of young children and videos with high levels of distortions were the hardest to annotate, even when aided with additional tool functionalities. Obtaining reliable RR measures through manual counting methods (direct and based on videos) in young (< 2 months) and agitated children is known to be very challenging and considered a bottleneck in creating reliable reference standards based on manual assessment of RR. Our results indicate that this tool cannot overcome these difficulties despite the option to annotate and remove distorted periods from RR calculation. This is especially challenging as the highest pneumonia burden occurs in younger children and ensuring that new devices perform adequately in this population is of highest priority.

As distortion levels seem to be highly correlated with levels of agreement, finding a way to objectively quantify distortion levels of a video independent of reviewers and prior to study commencement will be important for future studies. In the current study, level of distortion was computed as the average annotated distortion among the five reviewers and could have therefore been influenced by disagreement on presence and length of distorted periods between reviewers. The authors therefore welcome the recent initiative to create a quality-controlled open-access repository of annotated videos of children breathing for at least 60 s [9]. This could serve as an opportunity to explore methods to ensure sufficient quality of all videos included in the repository and to objectively tag videos with certain characteristics (e.g. low/high distortion levels), so future reference tools and diagnostic aids can be validated against a standardized and comparable set of features.

The overall SEM (4.8 bpm) observed in this study was larger than the conventionally accepted measurement error of ± 2 bpm [20]. At a WHO/UNICEF technical consultation in New York (October 2019), the scientific community acknowledged the difficulty of obtaining RR measures within these narrow limits and suggested that higher measurement errors should be considered sufficiently precise (K. Källander, 2019, personal communication, 7–9 October). While ‘the reality is that all reference device measurements include a degree of uncertainty’ as colleagues have previously pointed out [21], the challenge of defining cut-offs of agreement measures remains. While a *κ* of > 0.6 is considered substantial [22], do we consider this level of agreement substantial enough to establish a tool as a new reference standard for RR assessments or consider a new diagnostic aid sufficiently validated? Consensus on acceptable levels of measurement error and other measures of agreement needs to be reached in order to evaluate the quality of new tools and diagnostic aids.

As described, this study intended to obtain interrater measures of a new reference tool in ‘real-life’ settings to provide proficient evidence for this tool to serve as an adequate reference for new diagnostic aids claiming to provide reliable RR measures in these same settings. Real-life settings include instances where children are not calm and videos contain distorted periods making the annotation of breaths more challenging. However, we assumed that even automatic diagnostic aids would have difficulties obtaining reliable RRs beyond certain thresholds of distortion. Therefore, we did not include videos that contained levels of distortion deemed too high (> 30 s of distortion). This raises the question on how to deal with new diagnostic aids that claim to generate reliable RR measures under these circumstances as well. To our knowledge, neither the reference tool under investigation nor any other tool could currently serve as a reliable reference under these circumstances.

New diagnostic aids have moved beyond manual counting and have started to introduce new functionalities including the automatic removal of distorted or other uncertain periods and/or breath cycles. The formula to calculate RR defined as the number of breaths divided by the annotation period must be defined by the global community to take this progress into account. Currently, it remains unclear what type of breaths (certain, uncertain) and what time periods (completely calm, including uncertain areas) should be considered in the numerator and denominator of the RR formula. The current study considered only certain breaths and calm periods assuming close alignment with the IMCI guidelines and has described how distorted periods were defined and removed from the calculation. While the IMCI guideline offers a practical guide to calculating RR when using manual counting, it might not present the optimal way to calculate RR when additional elements (like distortions) can be taken into account. This issue goes beyond the objective of this paper, but should be part of a wider discussion among the scientific community on the computation of RR under consideration of these developments.

## CONCLUSION

We recommend that interrater agreement of the new reference tool in children younger than 2 months and agitated children needs to improve before it can be considered a new reference standard for validating new RR measuring diagnostic aids. Subsequent further evaluations of its inter- and intrarater agreement then need to be conducted. Obtaining a reference standard that shows good interrater reliability and agreement in all age groups younger than give years old and in children showing high levels of distortion remains crucial in order to reliably validate new diagnostic aids under real-life circumstances. Global consensus needs to be urgently documented to ensure that a global standard can be defined for RR reference tools going forward.
